# A Brief Review on Medicinal Plants-At-Arms against COVID-19

**DOI:** 10.1155/2023/7598307

**Published:** 2023-04-24

**Authors:** Shivani Srivastava, Fangzhou He, Yuanding Huang, Meng Niu, Alok Adholeya, Weng Kung Peng

**Affiliations:** ^1^Centre for Mycorrhizal Research, Sustainable Agriculture Division, The Energy and Resources Institute (TERI), TERI Gram, Gwal Pahari, Gurugram 122001, India; ^2^Songshan Lake Materials Laboratory, University Innovation Park, Dongguan 523-808, China; ^3^China Medical University, Shenyang, China

## Abstract

COVID-19 pandemic caused by the novel SARS-CoV-2 has impacted human livelihood globally. Strenuous efforts have been employed for its control and prevention; however, with recent reports on mutated strains with much higher infectivity, transmissibility, and ability to evade immunity developed from previous SARS-CoV-2 infections, prevention alternatives must be prepared beforehand in case. We have perused over 128 recent works (found on Google Scholar, PubMed, and ScienceDirect as of February 2023) on medicinal plants and their compounds for anti-SARS-CoV-2 activity and eventually reviewed 102 of them. The clinical application and the curative effect were reported high in China and in India. Accordingly, this review highlights the unprecedented opportunities offered by medicinal plants and their compounds, candidates as the therapeutic agent, against COVID-19 by acting as viral protein inhibitors and immunomodulator in (32 clinical trials and hundreds of *in silico* experiments) conjecture with modern science. Moreover, the associated foreseeable challenges for their viral outbreak management were discussed in comparison to synthetic drugs.

## 1. Introduction

COVID-19 (Coronavirus disease 2019) caused by novel SARS-CoV-2 virus is an ongoing pandemic responsible for the loss of more than 6.8 million as of February 2023 till date [1–3]. It manifests from mild illness (fever, cough, sore throat, and vomit) to severe respiratory illness (pneumonia) [4]. Multiplying its high infectivity, COVID-19 has imposed unprecedented threats to human health and global economy, leading to a catastrophic crisis of the world. Globally, exceptional efforts have been employed to control, treat, and reduce the impact and spread of SARS-CoV-2. As of 2023 (three years after the WHO announced COVID-19 as a pandemic), there are at least 18 approved vaccines under mass immunization drive (Table 1 [5–22]). Furthermore, Food and Drug Administration (FDA) special program known as Coronavirus Treatment Acceleration Program (CTAP) also reports a single agent (antivirals, cell and gene therapy, immunomodulators, and neutralizing antibodies) and combination agents for COVID-19 treatment with nine authorized for emergency use [23]. Antiviral investigational drugs such as remdesivir (Veklury) have received emergency use authorization (EUA) for COVID-19 treatment meeting eligibility requirements [24]. Immunomodulators such as dexamethasone have also been recommended for significant reduction in mortality in hospitalized patients (requiring oxygen or on ventilation) [25]. Though mass immunization drive, EUA drugs, convalescent serum, and monoclonal antibodies application are helping the world regain life normalization and confidence, there are certain high priority questions that remain unanswered in view of the longevity of the vaccine effect (requirement of booster doses; protection against mild to severe symptoms), impact of omission of long term safety assessment studies on frail, children and at-risk individuals, side effects, and above all loss in vaccine's potency against rapidly evolving and highly transmissible SARS-CoV-2 variants [26–29]. Issues related to effectiveness, dosage, and storage related to vaccines under trial for COVID-19 were also reported (Table 1). Thus, quest for finding a secure and efficient one-stop solution against SARS-CoV-2 and its variants will continue and we must be now prepared with the learning of our past experiences for what the future beholds.

Plant-based compounds have already been used to modulate the immunity and treat diseases of humans and other animals, which is an age-old method adopted over other medical sciences [30, 31]. Therefore, plant-derived compounds or phytochemicals have also been considered as potential compositions for the prevention or treatment of SARS-CoV-2. Current solutions against COVID-19 involve de novo drug/vaccine development that targets the human immune system or virus itself. Countering *de novo* drug/vaccine development, target-based drug repurposing/repositioning has emerged as a fast-track advantageous alternative in terms of established safety, low cost, and knowledge acquired from previous viral outbreaks [32, 33]. Medicinal plant-based traditional medicine has gained attention once again for utilization against the ongoing viral attack as evident from the increased demand of medicinal plants (e.g., ginger and turmeric) for boosting immunity in countries like the US and China [34]. Thus, in the absence of everlasting and safety issue of therapeutic modality for COVID-19, plant-based medicines (e.g., phytopharmaceuticals, plant derived secondary metabolites) explored and validated to show effective results in the past viral epidemic outbreak should be taken into prophylactic consideration.

## 2. Methods

**Figure 1:**
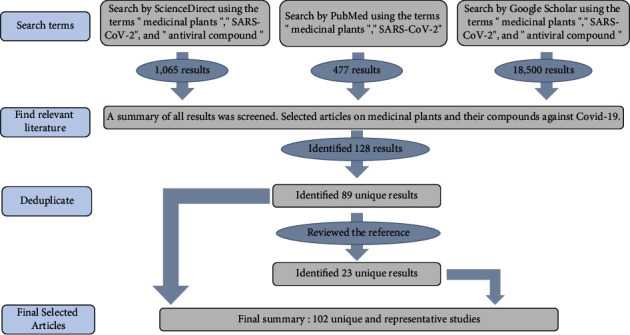
PRISMA diagram of the systematic literature review.

Papers published over the past three years (i.e., from January 1^st^, 2020, to February 2^nd^, 2023) in public databases, including ScienceDirect, PubMed, and Google Scholar, were searched by terms “medicinal plants,” “SARS-CoV-2,” and “COVID-19” (Figure 1). The published year was set to post-2020 to ensure their novelty, and the results were limited to the top 50 on ScienceDirect, PubMed, and Google Scholar to guarantee their relevance. Then, the abstracts of each article were reviewed to assess if the articles were relevant to the topic. The references of each article were also gone over in order to find additional technical approaches. Based on abovementioned inclusion criteria, the most appropriate articles were selected for review and discussion. For each article and case study included, were classified into 3 categories: (1) reported methodology; (2) target sites; and (3) plant-based antiviral compounds (Table 2).

### 2.1. Status on Registered Clinical Trials with Plant-Based Medicines and Drug Supplements

Over 8800 studies related to COVID-19 of clinical trials were officially reported in ClinicalTrials.gov, including 32 studies based on plant-derived compounds. To provide an overview on drug supplements, with herbal and botanicals and plant-derived secondary metabolites candidates under clinical trials as the therapeutic agent against COVID-19, we mapped and analyzed two registration databases (i.e., the World Health Organization's International Clinical Trials Registry Platform (ICTRP) and ClinicalTrials.gov). ClinicalTrials.gov listed privately and publicly funded clinical studies conducted around the world [61]. Similarly, ICTRP is a central database containing the trial registration data submitted by the sixteen listed registries in the WHO [62]. Data from ICTRP were collected on 24^th^ Sept 2020 and from Clinical Trials on 29^th^ Sept 2020.

**Figure 2:**
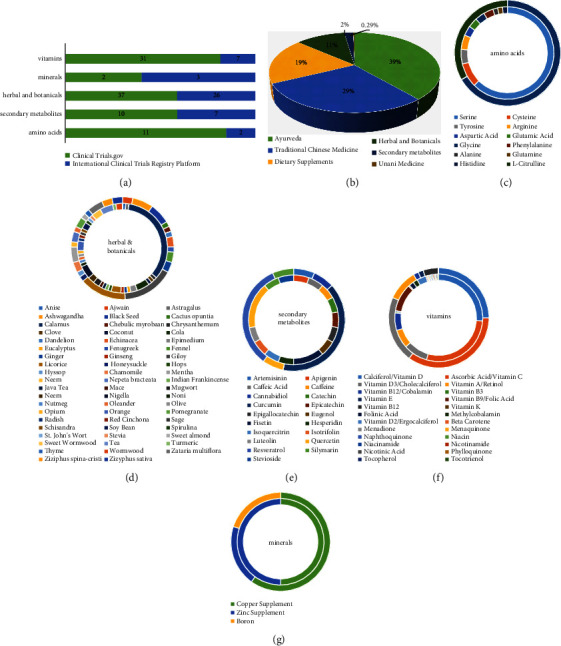
Schematic representation of registered clinical trials for COVID-19 on drug supplements, herbal and botanicals, and plant-derived secondary metabolites. Data collected from two registry platforms (ClinicalTrials.gov and ICTRP). (a) Bar chart representing number of clinical trials registered in ClinicalTrials.gov and ICTRP for vitamins, minerals, herbal and botanicals, secondary metabolites, and amino acids, (b) pie chart showing analysis of data collected from ICTRP on the percentage share of Ayurveda (39%), traditional Chinese medicine (29%), dietary supplements (19%), herbal and botanicals (11%), secondary metabolites (2%), and Unani medicine (0.29%) in registered clinical studies apart from vaccines. Doughnut representation showing individual category on (c) amino acids, (d) herbal/botanical, (e) secondary metabolites, (f) vitamins, and (g) minerals registered for clinical trials at ClinicalTrials.gov (inner doughnut) and ICTRP (outer doughnut).

ClinicalTrials.gov classifies registered COVID-19 studies under dietary supplements category as amino acids, flavonoids, herbal and botanicals, minerals, other dietary supplements, and vitamins. For our study interest and comparative analyses of ClinicalTrials.gov with ICTRP, we reclassified the data into five major categories of amino acids, herbal and botanicals, secondary metabolites, vitamins, and minerals as shown in Figure 2(a). Studies registered under herbal and botanicals (63) and plant derived secondary metabolites (17) [63] support the curative potential of plant-based medicine against COVID-19. Furthermore, detailed analyses of ICTRP data also revealed traditional medicine (Ayurveda; 39% and Traditional Chinese Medicine (TCM); 29%) related studies registration (Figure 2(b)). Landscaping of five reclassified categories was also performed to showcase categories and their registration count (Figures 2(c)–2(g)). Specifically, there were 12, 59, 19, 24, and 3 clinical trial studies registered on amino acids, herbs and botanicals, secondary metabolites, vitamins, and minerals, respectively. Medicinal plants such as Giloy (*Tinospora*), licorice (*Glycyrrhiza*), and calamus (*Acorus*) and secondary metabolites such as quercetin, resveratrol, and curcumin were observed as major candidates in the registered studies. Similar to plant-related candidate's vitamin C and D, serine and cysteine and copper supplements were the other major players. Fifty-nine herbals/botanicals under clinical trial studies corroborates exploration of plant-based medicines against COVID-19 and its related impacts.

### 2.2. Medicinal Plants and Their Curative Potential against COVID-19

It has accumulated thousands of years of experience in the treatment of epidemic diseases with medicinal plants. Moreover, inspired by the treatment of SARS Coronavirus (SARS-CoV) in late 2002 [64], they were reasonably considered for the treatment of SARS-CoV-2. It has shown that the SARS-CoV mortality rate in Beijing dropped from 52% to 2% in one month with the adjunctive treatment of medicinal plants in China [65]. Traditional medicines practiced throughout the world such as “Traditional Chinese Medicine” or “Indian Ayurveda” or “Egyptian pharmacopeia” has always relied on the medicinal plants for their therapeutic potential. Early nineteenth century witnessed the initiation of modern pharmaceutical industry with a strong presence of medicinal plants evident from their one-fourth share in new molecular entities/drugs from natural resources followed by FDA approval [66]. In June 2020, Chinese government in a White Paper declared the unique strength of medicinal plants for COVID-19 treatment and control *via* preemptive prevention, differentiated medication, and multitargeted intervention with more than 90% effectivity in Hubei province that received TCM treatment [67]. Similarly, Indian traditional medicine Ayurveda also holds methodologies for cure of respiratory illness through local and systemic interventions involving utilization of herbal decoctions/medicated water, etc. [68]. Herbal decoctions/polyherbal tablets utilized in Ayurvedic or TCM are combinatorial preparations that play a role in immunity stimulation or as inflammation-modulating agents or as antiviral agents for COVID-19 control and prevention [34, 69, 70]. Ayurvedic medicines such as Indukantham Kwatham, Vilvadi Gulikaand, and Mukkamukkatuvadi Gulika recommended for use by patients with mild symptoms in COVID-19 are combinations of 16, 13, and 21 different medicinal plants, respectively [70]. Similarly, three decoctions namely Qingfei Paidu, Huashi Baidu, and Xuan Fei Baidu decoctions promoted for use in China against COVID-19 are combinations of 21, 14, and 13 different medicinal plants, respectively [34].

Curative potential of these traditional pharmacopeias can be ascribed to active ingredients (secondary metabolites) produced by plants. Medicinal plants are reservoirs of secondary/specific metabolites (flavonoids, alkaloids, glucosides, and polyphenolics) that have antioxidant, bacterial, viral, fungal, cancer, tumor, diabetic, and malarial like properties [32, 33, 66]. Their extracts have been extensively screened for drug molecules to evaluate *in vitro* antiviral activities [32]. Crude extracts obtained from *Lycoris radiata*, *Artemisia annua,* and *Lindera aggregate* and the pure natural products isolated from *Isatis indigotica*, *Torreya nucifera,* and *Houttuynia cordata* have shown anti-SARS effects and identified as potential candidate for the treatment of two Coronavirus epidemic outbreaks (MERS-CoV in 2012 and SARS-CoV in 2013), seasonal influenza virus epidemics, and dengue virus [32, 71–74].

**Figure 3:**
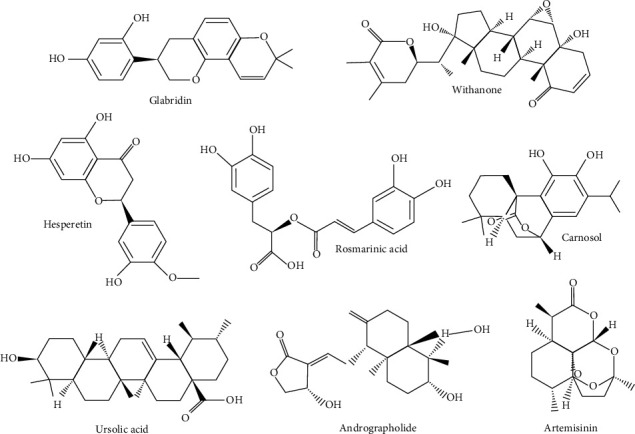
Potential plant-derived secondary metabolites having anti-SARS-CoV-2 activity.

Keeping plant's medicinal properties in view, research in the field of molecular docking to identify potential anti-SARS-CoV-2 compounds has grown immensely against target sites of COVID-19 proteases, human angiotensin-converting enzyme, RNA dependent RNA polymerase, glycoprotein inhibitors, angiotensin-converting enzyme 2, and others [35–60]. Table 2 shows a list of potent anti-SARS-CoV-2 compounds identified by molecular docking studies, their target sites, and plant resources. Medicinal plants such as *Glycyrrhiza*, *Withania*, *Ocimum*, *Tinospora*, and *Andrographis* have emerged as prospective candidates for anti-SARS-CoV-2 activity. Secondary metabolites such as coriandrin, glabridin, glucobrassicin, ursolic acid, apigenin, oleanolic acid, rosmarinic acid, vallesiachotamine, hypericin, baicalin, andrographolide, myricitrin, silybin, scutellarin, hesperetin, glycyrrhizin, epigallocatechin, and withanone are reported as prominent active ingredients (also shown in Figure 3).

### 2.3. The Effect of Medicinal Plants on SARS-CoV-2

**Figure 4:**
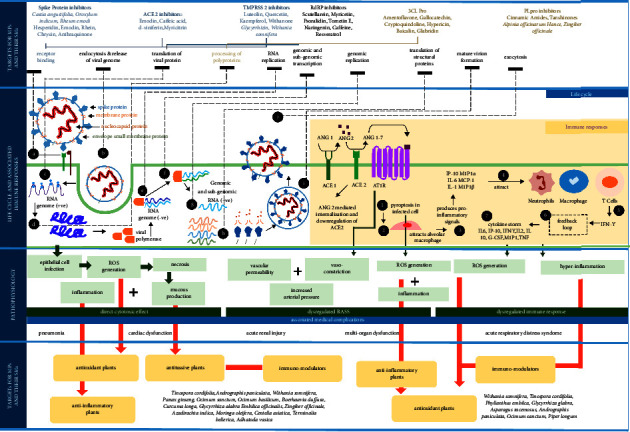
Life cycle of SARS-CoV-2, associated immune, and pathophysiological responses for identifying the targets and role of medicinal plants and their secondary metabolites against COVID-19.

It was found that the medicinal plants effectively inhibited SARS-CoV-2 in five aspects: life cycle, immune responses, associated pathophysiology, role of medicinal plants, and their secondary metabolites. We have summarized the stages involved in the life cycle of SARS-CoV-2 followed by immune response developed by the host cell upon viral infection and development and associated pathophysiological response that lead to medical complications to identify the potential targets for action by medicinal plants and their secondary metabolites [75–78] (Figure 4). Basically, the life cycle of SARS-CoV-2 involves ten steps from (i) viral entry through the binding of spike (S) protein to ACE 2 (angiotensin-converting enzyme (2) receptor and priming of S protein by cellular serine proteases (TMPRSS2), (ii) membrane fusion; endocytosis and viral genome release, (iii) translation of viral protein, (iv) processing of viral polyproteins, (v) RNA replication, (vi) genomic and subgenomic transcription, (vii) genomic replication, (viii) translational of structural proteins, (ix) the formation of mature virion, and (x) its release via exocytosis. Medicinal plants and their secondary metabolites can act as spike protein, ACE2, TMPRSS 2, RdRp, 3CL Pro, and PL pro inhibitors. Synchronous to the viral life cycle progression, the host cell undergoes pryoptosis and releases damage associated signals that triggers generation of proinflammatory cytokines and chemokines leading to inflammatory responses and ultimately to creation of proinflammatory feedback that causes medical complications such as pneumonia, cardiac dysfunction, acute renal injury, multiorgan dysfunction, or acute respiratory distress syndrome. Medicinal plants having antioxidant, antitussive, anti-inflammatory, and immunomodulatory properties can be utilized for ailments of physiological response generated by SARS-CoV-2. Direct virus-mediated cytotoxic effect, dysregulation of the RAAS (renin-angiotensin-aldosterone system) due to downregulation of ACE2 related to decreased cleavage of angiotensin I (ANG1) and angiotensin II (ANG 2) and dysregulation of the immune response and hyper-inflammation can be proposed as mechanisms for COVID-19 [76, 79].

In China, the application of TCM has been acclaimed for COVID-19 prophylaxis. Through both the network pharmacological and the literature review, Huang et al. [33] recently summarized the top five ingredients, targets, and potential mechanisms underlying the actions of TCM (medicine/decoctions). They reported the role of TCM on viral protein binding and replication, suppression of cytokine storm, alleviation or elimination of excessive immune response, and organ protection. COX-2, CASP3, IL-6, MAPK1, MAPK14, MAPK8, and RELA were identified as top five targets in the their analyses followed by IL-17, arachidonic acid metabolic pathway, HIF-1, NF-*κ*B, Ras, and TNF as the top five prospective signaling pathways.

### 2.4. In Silico Approach: Traditional Active Compounds Can Act as Immunomodulators against SARS-CoV-2

With the tremendous progress of computer power and artificial intelligence, *in silico* approaches have been broadly adopted in the treatment of pandemic health emergencies. Sahoo et al. [80] systematically tested the potential combinations in anti-HIV drugs and vitamin C derivatives through bioinformatic tools, which implied possible less toxic regimens for the treatment against COVID-19. There is an urgent need for the involvement of an *in silico* approach in pandemic health emergencies. Chen and Du [42] concluded that baicalin, hesperidin, nicotine, and glycyrrhizin have the potential to bind to ACE2 and block the entry of COVID-19. Rudrapal et al. [81] identified six bioactive phytomolecules with a promising anti-SARS-CoV-2 potential by *in silico* screening. Bharathi et al. [82] identified five compounds: acteoside, verbascoside, kanzonol V, progeldanamycin, and rhodoxanthin, which acted effectively against the target residues in the receptor-binding domain (RBD) and significantly against the Indian delta variant of COVID-19 compared with ceftriaxone. Raugh studied 65 bioactive compounds in tea leaves to provide a lead compound for NSP16 against SARS-CoV-2. Through in silico (molecular dynamics simulation) analysis, Singh et al. [83] found that flavin compounds exhibited lower binding free energy than the standard molecule sinefungin.

### 2.5. Challenges and Opportunities

Synthetic drugs are often associated with treating disease symptoms and not the root cause. In fact, no less than 100,000 people each year die due to these toxicities and about 8% of hospital admissions in the United States of America are due to adverse or side effects of synthetic drugs. On the contrary, deaths or hospitalizations due to herbs are so rare that the National Poison Control Centers of the United States does not have a database for side or adverse effects of herbs. Medicinal plant mechanisms are often complicated and not straightforward [84]. Compared to the synthetic inhibitors, plant-based drugs have less toxicity and are much safer to use. It has been demonstrated that a combination of isolated active compounds is not able to mimic the whole plant effects. This is perhaps the reason why medicinal plants usually tend to have several broad complementary or synergistic actions on physiological systems simultaneously, usually in the same general therapeutic direction and often nonspecific.

COVID-19 remains a new disease with limited information available on its pathophysiology. Thus, prospective plant-based drug molecules must be analyzed *in silico*, *in vitro* (cell lines based assays), and *in vivo* (animal models) before clinical trials with a strong pharmacokinetic profile to address issues related to their efficacy potential and poor bioavailability. Di Matteo et al. [85] argued that the characterization of potential drug candidates through computer studies can improve the efficiency of clinical trials. Furthermore, to amplify their chances towards qualification for designation as a drug molecule, novel formulation(s) must be developed in combination analyzed for their administration. Key attractions of plant-based medicines lies in their low cost, prospects for scalability, and safety; however, issues in maintenance of the quality of raw material, limited information on mechanism of action, and efficacy due to multiple components are the bottlenecks. Plant-based medicines offer to developing countries that have limited resources to cope up with the current pandemic situation. Similarly, this situation also exists with developed countries; however, reformation/amendments in their existing regulatory policies are required. Further facilitation and incentive for interdisciplinary research in the field of medicinal plants are also required. To highlight the current strength, weakness, opportunities, and challenges of medicinal plant vs. synthetic drugs against COVID-19, we have represented a summary of the short analysis in a SWOT format (Table 3).

### 2.6. The Hidden Hands of Asymptomatic Subjects

It is estimated that 1 in 4 of the corona patients may be asymptomatic [86]. The finding suggests that isolation of symptomatic subjects alone is futile and will not stop the ongoing spread of SARS-CoV-2. One of the main tasks in pandemic control and elimination would therefore be to invest in the identification of the largely undetected asymptomatic subjects (and hence the huge reservoirs of the virus). Therefore, “false negative” cases are not uncommon, given that the biomarkers (e.g., antigen or antibody) may fall beyond the thresholds of the limit-of-detection of each technology. Current diagnostic approaches fall largely into two categories, i.e., polymerase chain reaction (PCR) and rapid antibody test (RDT) based. First, the PCR antigen test which detect the presence of the viral acid nucleic in the body. Second, the RDT is to detect the early immune response to the infection. If a subject is infected, their blood will contain antibodies to the virus. However, none of this is ideal, with the PCR and RDT suffering from long procedural time and specificity problems, respectively. Mass screening is the expensive way to go and may seem to be the only way. It has been done multiple times in China. Qingdao, for example, is testing its entire population of nine million people for COVID-19 over a period of five days. Wuhan was earlier reported to have tested over nearly 11 million of its entire population [87]. On the other hand, the devil's advocate may propose running a medicinal plant diets campaign rendering the needs for mass screening instead (or at least as complementary measure). This may be one of the acid tests placed highly in postlockdown pandemic management.

### 2.7. The Throne of Personalized Medicine

One of the Holy Grails to speed up the translational use of medicinal plants is through the active employment of modern spectrometers to check on the herbal authenticity (or otherwise its toxicity) and the metabolism of the herbs in a highly regulated and reproducible manner. Not until the 1960s, structural elucidation of natural products used to be a time-consuming complicated process requiring technical skill. It relied almost entirely on degradative chemistry or elemental analysis of the crystallized material. With the advent of spectroscopic techniques (e.g., nuclear magnetic resonance (NMR), ultraviolet, and infrared) and mass spectrometry, new structural biology is solved typically within hours (if not days).

Of those four modern techniques, NMR is the most rapid (in minutes), robust, and relatively high sensitivity, requiring only submilligram quantities of amorphous products [56]. It provides detailed information both on the frequency domain (e.g., chemical shift) [88] and time domain (e.g., relaxometry) [88, 89] from a whole range (library of pulse sequences) of highly informative two- and multi-dimensional spectra. 2D NMR-based metabolomic (coupled with multivariate analysis) can be used to extract information on targeted compound analysis, metabolic profiling, metabolomics fingerprinting, and metabolic analysis.

It is worth noting that the administration of active compounds (in the form of pure chemical) and that of a plant extract containing the same chemical entity is essentially different in their bioavailability as well as mode-of-actions [90]. Humans are faced with the reality that life forms and living organisms are in general complex. Thus, the rise of functional assays (e.g., functional genomics, functional proteomics) in the era of postgenomics is not an unexpected one.

Recent reports on functional phenotyping using low-field NMR-based micro scanner (e.g., malaria [90–93], diabetes mellitus, hemoglobinopathies, and trauma-emergency) on live biological cells/tissues (*ex vivo*) obtained directly from patients open up new opportunities in *in vitro* diagnosis in personalized manner [94–97]. NMR-based microscanner is the scaled down version of a large and expensive MRI machine which is being used clinically to map out the human body noninvasively. The same noninvasive technology can be used to map a single drop of blood using much simpler and compact hand-held instrumentation [98]. Miraculously, a single drop of blood contains enormous amounts of untapped complex information (and yet patient-specific and time-unique) at the molecular level akin to unique molecular QR-code (or “molecular fingerprint”) [95] as the attribute of various physiological and pathological states. The complex signatures were extracted using two- and multi-dimensional time-domain NMR correlation spectroscopy and deciphered with the help of machine learning for disease classification [95, 99, 100] (e.g., diagnosis and prognostic) is making fast in-road into medical research and the clinical translational stage [99, 100].

### 2.8. Recent Development of Medicinal Plant in the Post-Pandemic Era

As the governments around the world have liberalized the control of the pandemic, great outbreak seems have pasted. However, there are still possibilities of another virus mutation and consequent widespread infection. Strenuous efforts are still of need for seeking the treatment method, including the medicinal plant-based way.

According to TCM, the clinical manifestations of COVID-19 infections fall into two categories, one being respiratory symptoms and the other being digestive symptoms. During the recovery period, if the patient's lung and spleen functions have not fully recovered, the patient will suffer from weakness and shortness of breath; if the patient has a deficiency of the spleen and liver wood, the patient will suffer from insomnia. Furthermore, there will be a legacy of taste and smell disorders if the functions of the lungs and spleen of the patients are not fully restored, as the lungs are associated with the nose and the spleen with the mouth. Abovementioned has shown that treatment during the recovery period is mainly directed at the two organs, the lung and the spleen; thus, the clinical treatment can be based on formulas that promote the lung, moisten the lung, strengthen the spleen, and resolve dampness. Luo et al. [65] stated that the most frequently used medicinal plant included Radix astragali (Huangqi), Radix glycyrrhizae (Gancao), and Radix saposhnikoviae (Fangfeng). In South America, Arun Dev Sharma found eucalyptus (*Eucalyptus globulus* Labill.) to be an effective antiviral agent against SARS-CoV-2. Janenone, a vital essential oil from *Eucalyptus globulus* oils, can penetrate nonspecifically into the lipid bilayer of the virus and alter the fluidity of the membrane and has antiviral effects against the main protein of SARS-CoV-2 [101, 102]. Safa et al. [103] administered a 1000 mg dose of ginger tablets three times a day for three months to COVID-19 infected patients and found clinical improvement in symptoms including fever, dry cough, and malaise.

## 3. Conclusion

In short, COVID-19 outbreak is unfortunately not the first viral outbreak or the last one. This pandemic which came (and went) like a storm had left many of us pondering about the fate of humanity with respect to disaster control. Can we ever learn to live in harmony with Nature? Or made to eat the humble pie in the name of economic progress? Medicinal plants have been in existence as long as human beings have existed on this planet earth. It is said that there is a natural cure for every disease on earth. We humbly appeal to the authority, researcher, and society at large to look seriously into the huge potential of medicinal plants and their secondary metabolites. With intensified effort to develop this platform into evidence-based medicines, what was once an “alternative medicine” can work in synergy with “main-stream medicine.” Human society can utilize current COVID-19 adversity as a window of opportunity for change and an hour to adapt plant-based medicine for a new era of sustainable health.

## Figures and Tables

**Table 1:** Vaccines under trial for COVID-19, their types, effectiveness, dosage, and storage.

Companies	Country	Name	Type	Trials	Approval	Effective (%)	Dose/gap period (days apart)	Storage/duration (days/weeks)	Reference
FBRI	Russia	EpiVacCorona	Peptide antigens-based	03	01	100	2X/21–28 d	2° to 8°C	[5, 6]
Moderna	USA	mRNA-1273	Lipid nanoparticle (LNP)-encapsulated mRNA	07	40	94.1	2X/28–42 d	2° to 8°C/30 d	[7, 8]
Pfizer/BioNTech	Germany USA	BNT162b2	Lipid nanoparticle (LNP) formulated, nucleoside modified RNA vaccine	12	68	95	2X/21 d	−25°C to −15°C/2 weeks	[9]
CanSino	China	Ad5-nCoV	Nonreplicating viral vector	06	032	65.7–91%	Single dose	2° to 8°C	[10]
Gamaleya	Russia	Sputnik V	Nonreplicating viral vector	19	45	91.6–100	2X/21 d	2° to 8°C	[11]
Janssen (Johnson & Johnson)	USA	Ad26.COV2.S	Nonreplicating viral vector	08	04	66–85	Single dose	2°to 8°C/3 months	[12]
Oxford/AstraZeneca	UK	AZD1222	Nonreplicating viral vector	22	74	81.3	2X/12 weeks	2° to 8°C/6 months	[13]
Serum Institute of India	India	Covishield	Nonreplicating viral vector	02	17	81.3	2X/12 weeks	2° to 8°C/6 months	[13]
Bharat Biotech	India	Covaxin	Inactivated	0	03	81	2X/21 d	2° to 8°C	[14]
Sinopharm (Beijing)	China	BBIBP-CorV	Inactivated	06	20	79	2X	2° to 8 °C	[15]
Sinopharm (Wuhan)	China	Inactivated (vero cells)	Inactivated	06	02	72.51	2X	2° to 8°C	[16]
Sinovac	China	CoronaVac	Inactivated	13	16	50.4–100	2X	2° to 8°C	[17]
AstraZeneca	Sweden	Vaxzevria	mRNA vaccine	03	121	74	2X/12 weeks	2° to 8°C	[18]
Novavax	USA	Nuvaxovid	Recombinant protein vaccine	03	—	90.4	2X/8 weeks	2° to 8°C	[19]

**Table 2:** List of plant-based anti-SARS-CoV-2 compounds identified through *in silico* approaches.

Reported methodologies	Target sites	Plant-basedantiviral compounds	Reference
Molecular docking	(i) Covid-196LU7 proteases (ii) Covid-196Y2E proteases	(i) Coriandrin (ii) Glabridin (iii) Glucobrassicin (iv) Ursolic acid (v) Hederagenin (vi) Apigenin (vii) Oleanolic acid (viii) Rosemarinic acid	[35]
*In silico* screening molecular docking DFT molecular modelling	(i) COVID-19 proteases	(i) Oleanic acid (ii) Ursolic acid (iii) Isovallesiachotamine (iv) Vallesiachotamine (v) Cadambine (vi) Vincosamide-N-oxide (vii) Isodihydroaminocadambine (viii) Pentyle ester of chlorogenic acid (xi) D-myo-inositol	[36]
Molecular docking using autodock vina and GOLD suite Cys145 and His41 residues quantitative structure-activity relationships (QSAR)	(i) 3-C like protease	(i) Hypericin (ii) Cyanidin 3-glucoside (iii) Baicalin (iv) Glabridin (v) Α-ketoamide-11r	[37]

Molecular docking target analysis and toxicity prediction ADME prediction	(i) SARS-CoV-2 main protease	(i) Andrographolides	[38]

*In silico* analysis	(i) Human angiotensin-converting enzyme 2 (hACE-2) (ii) RNA dependent RNA polymerase (RdRp)	(i) D-viniferin (ii) Myricitrin (iii) Taiwanhomoflavone A (iv) Lactucopicrin 15-oxalate (v) Nympholide A (vi) Afzelin (vii) Biorobin (viii) Hesperidin (ix) Phyllaemblicin B	[39]
Molecular docking	(i) SARS-CoV-2 M^pro^	(i) Rosmanol (ii) Carnosol (iii) Arjunglucoside-I	[40]

*In silico* analysis	(i) Higher binding affinity with targets	(i) Silybin	[41]

*In silico* analysis	(i) Angiotensin-converting enzyme 2	(i) Baicalin (ii) Scutellarin (iii) Hesperetin (iv) Nicotianamine (v) Glycyrrhizin	[42]

*In silico* analysis	(i) Glycoprotein inhibitors	(i) Hesperidine (ii) Cannabinoids (iii) Pectolinarin (iv) Epigallocatechin gallate (v) Rhoifolin	[43]

*In silico* analysis	(i) SARS-CoV-2 mpro	(i) Bonducellpin D (ii) 5, 7-dimethoxyflavanone-4′-O-*β*-d-glucopyranoside (iii) Caesalmin B	[44]

Molecular docking	(i) SARS-CoV-2 mpro	(i) B-eudesmol (ii) Digitoxigenine (iii) Crocin (iv) a-Terpeneol (v) b-Carophyllene (vi) Picrocrocin (vii) Crocetin (viii) Calarene (xi) Bicyclogermacrene	[45]

Molecular docking	(i) SARS-CoV-2 3CL^pro^	(i) 10-hydroxyusambarensine (ii) Cryptoquindoline (iii) 6-oxoisoiguesterin (iv) 22-hydroxyhopan-3-one	[46]

Molecular docking	(i) SARS-CoV-2 3CL^pro^ (ii) Plpro (iii) RdRp (iv) Spike protein	(i) Chloroquine (ii) Luteolin	[47]

Molecular docking	(i) SARS-CoV-2 3CL^pro^	(i) Luteolin (ii) Licoisoflavone B (iii) Fisetin (iv) Quercetin (v) Glyasperin F (vi) Isolicoflavonol (vii) Semilicoisoflavone-B	[48]

Molecular docking	(i) Covid 19 protein 6W63	(i) Narcissoside	[49]

Molecular docking	(i) SARS-CoV-2 M^pro^	(i) Theaflavin digallate	[50]

Molecular docking	(i) SARS-CoV-2 S-protein	(i) Phillyrin (ii) Chlorogenic acid	[51]

Molecular docking	(i) GRP78 (ii) 6LU7 (iii) SARS-CoV-2 M^pro^	(i) Withaferin A (ii) Artemisinin (iii) Curcumin (iv) Andrographolide	[52]

Molecular docking	(i) SARS-CoV-2 M^pro^	(i) Oolonghomobisflavan-A (ii) Theasinensin-D (iii) Theaflavin-3-Ogallate	[53]

Molecular docking	(i) SARS-CoV-2 M^pro^	(i) Ursolic acid (ii) Carvacrol (iii) Oleanolic acid	[54]

Molecular docking	(i) SARS-CoV-2 mpro (ii) SARS-CoV-2 Nsp15/NendoU (iii) SARS-CoV-2 ADRP (iv) SARS-CoV-2 RdRp (v) SARS-CoV-2 rS (iv) HACE2	(i) (E)-*β*-farnesene (ii) (E, E)-*α*-farnesene (iii) (E)-*β*-farnesene, (iv) (E, E) farnesol	[55]

Molecular docking	(i) SARS-CoV2 E	(i) Belachinal (ii) Macaflavanone E (iii) Vibsanol B	[56]

Molecular docking	(i) Angiotensin-converting enzyme 2	(i) Isothymol	[57]

Molecular docking	(i) RdRp	(i) Theaflavin	[58]

Molecular docking	(i) SARS-CoV-2: ACE2	(i) Dithymoquinone	[59]

Molecular docking	(i) SARS-CoV-2 mpro	(i) Luteolin-7-glucoside (ii) Demethoxycurcumin (iii) Apigenin-7-glucoside (iv) Oleuropein (v) Curcumin (vi) Catechin (vii) Epicatechin-gallate	[60]

**Table 3:** SWOT analysis of medicinal plant vs. synthetic drugs against COVID-19.

Medicinal plants			Synthetic drugs	
Strengths	(i) Broad-spectrum, (ii) Holistic-integrating emotional, mental, and spiritual aspects	(i) Long historical records	(i) Mechanism of active compound known, targeted	(i) Stringent *in vivo* studies

Weakness	(i) Quality maintenance (ii) Raw material adulteration issues (iii) Possible toxic effect (if not well prepared) (iv) Efficacy due to multiple ingredient/not single molecule dependent	(i) Mechanism complicated (or unknown) (ii) Bioavailability and true efficacy in vivo	(i) (Long-term) side effect/s	(i) Short history (>200 years)

Opportunities	(i) Second opinion (ii) Low-cost production, free to use, less side effect (iii) Amenable for genetic modifications for engineering plant metabolites can be helpful for developing a specific drug (iv) Prospects for scalability and safety	(i) Antioxidant properties for chronic diseases, off-shelf remedies (ii) Preservation of medicinal plant biodiversity in the world (iii) Opportunity to developing countries (iv) Oral formulation development	(i) Infrastructures available (ii) Immediate gains	(i) Industries ready to absorb the drug for sale

Challenges	(i) Quantification (dosage-not well defined) (ii) Development of formulations for better bioavailability	(i) Harmonization to western medicine (ii) Limited funds availability (iii) Regulations (iv) Skilled manpower	(i) New drugs development and validation take years	(i) Dominant by big pharma

## Data Availability

Data used to support the findings of this study are available upon reasonable request from the corresponding author.
